# Design and study of nitric oxide portable producing device using continuous discharging arc plasma reaction keeping low energy efficiency for viral pneumonia emergency therapy

**DOI:** 10.1371/journal.pone.0237604

**Published:** 2020-08-13

**Authors:** Li Qian, Zhang Wenlu, Wang Hong, Deng Juan, Tian Xinli, Wang Linlin, Sha Hong

**Affiliations:** Institute of Biomedical Engineering, Chinese Academy of Medical Sciences & Peking Union Medical College, Tianjin, China; Missouri University of Science and Technology, UNITED STATES

## Abstract

This study investigated the efficiency of a portable nitric oxide (NO) inhalation device through optimizing its design and structure. The portable rescue device could be used in clinical applications in outbreaks of viral pneumonia such as SARS. To reduce energy consumption for battery-powered portable usage, NO micro-channel plasma reactions induced by a continuous discharge arc were employed. A single-use airway tube could be combined with an intubation tube in clinical applications. In the experiment, a switching transistor controlled high frequency DC (12.5 kHz) was used to create a continuous discharge arc between two stainless steel electrodes (1-mm separation) after high-voltage breakthrough. A rotate instrument was employed to change the direction angle between the airflow and discharge arc, tube filled with Calcium hydroxide connected with gas outlet for reducing NO_2_, gas flow rate and input voltage were evaluated separately with concentration of NO and NO_2_/NO ratio. Results showed that a 2 L/min air flow direction from the cathode to the anode of electrodes (direction angle was zero) under 4 V input voltages produced 32.5±3.8 ppm NO, and the NO_2_/NO ratio reduced to less than 10%, stable output of nitric oxide might be convenient and effective for NO inhalation therapy. Modularization of the design produced a portable NO inhalation device that has potential for use in clinical applications as it is low cost, easy to disinfect, consumes low levels of energy and is ready to use.

## Introduction

Nitric Oxide (NO) which has attracted widespread attention from researchers in the medicine and biology fields, is a chemically unstable gas that is easily oxidized to Nitrogen dioxide (NO_2_). NO is a new type of bio-messenger molecule that has been identified as the endothelium-derived relaxing factor (E-DRF) that can relax blood vessels in the organism [1]. Inhalation of NO at concentrations between 3 and 20 ppm can selectively expand the pulmonary arteries and improve the oxygenation function of the lungs [2, 3]. NO inhalation therapy has achieved positive effects in the treatment of various cardiopulmonary diseases such as pulmonary hypertension and chronic obstructive pulmonary disease [4–6]. Akerstrom et al. [7, 8] found that NO inhibits RNA replication and affected S protein palmitoylation. As NO may have a dual effect on SARS-CoV treatment through potentially improving the oxygen intake in the lungs and suppressing the coronavirus, it is helpful for the treatment of critically-ill patients in clinical settings prior to intubation treatment.

At present, medical NO is produced by chemical methods such as redox reaction by NaNO_2_ and H_2_SO_4_, and stored in high-pressure cylinders at concentrations of 500–800 ppm. NO therapy devices include a gas cylinder and a gas supply system, which are used in combination with a ventilator. The concentration of NO, NO_2_, and O_2_ is adjusted by a complex delivery device through multiple steps of decompression and dilution [9]. This therapy device requires high levels of power, and is an expensive and sophisticated therapeutic instrument that requires professional operators and trained respiratory therapy staff. Patients need to inhale a certain amount of NO in a fixed position, which limits its use. Due to these disadvantages, NO inhalation therapy is rarely applied in a wide range of application scenarios, and has proved difficult to popularize in the treatment of chronic diseases and relief of sudden symptoms. Therefore, it is necessary to develop a simple, portable and economical NO inhalation treatment system.

NO could be produced in a thermal equilibrium plasma during arc reaction generated by pulsed discharges in the air. Many researchers have made many attempts to generate medical NO by pulsed discharge. Namihira et al. [10–12] carried out much research on conditions for NO production in pulsed discharges, including using electrode materials, the discharge method, gas flow rate, NO_2_ removal devices and other experimental conditions. Hui et al. [13] studied the chemical reaction mechanism of NO produced by N_2_ and O_2_. They studied the changing trend of arc temperature from a micro perspective to control the discharge process and find the optimal output mode for NO_2_/NO ratio decreases. Yu et al. [14] designed a portable NO generation system, which was still in the research stage and no mature technology application. Pulsed arc discharge with a pulse frequency lower than 5 kHz was used to produce NO in Namihira, Hui and Yu’s studies. In general, air dielectric strength could be basically restored in only 200 μs after being penetrated. In the process of low-frequency pulse discharge, air needs to be repeatedly penetrated, which consumes a lot of energy. To date it has been difficult to develop a small, efficient, and low-power portable NO rescue device by using low-frequency pulse discharge.

In this study, a low-power portable NO rescue device was designed and developed that producing NO using the high-frequency pulsed arc discharge method. By using high-frequency pulses, the air between the electrodes was much more easily penetrated, which achieved the goal of developing a way of producing of NO with low power consumption and high efficiency. We optimized the structure of the device and maintained the continuous plasma reaction condition to generate a stable and suitable concentration of NO for inhalation therapy, which made it possible to inhale NO in any position and location.

## Methods

### System design

**Fig 1:**
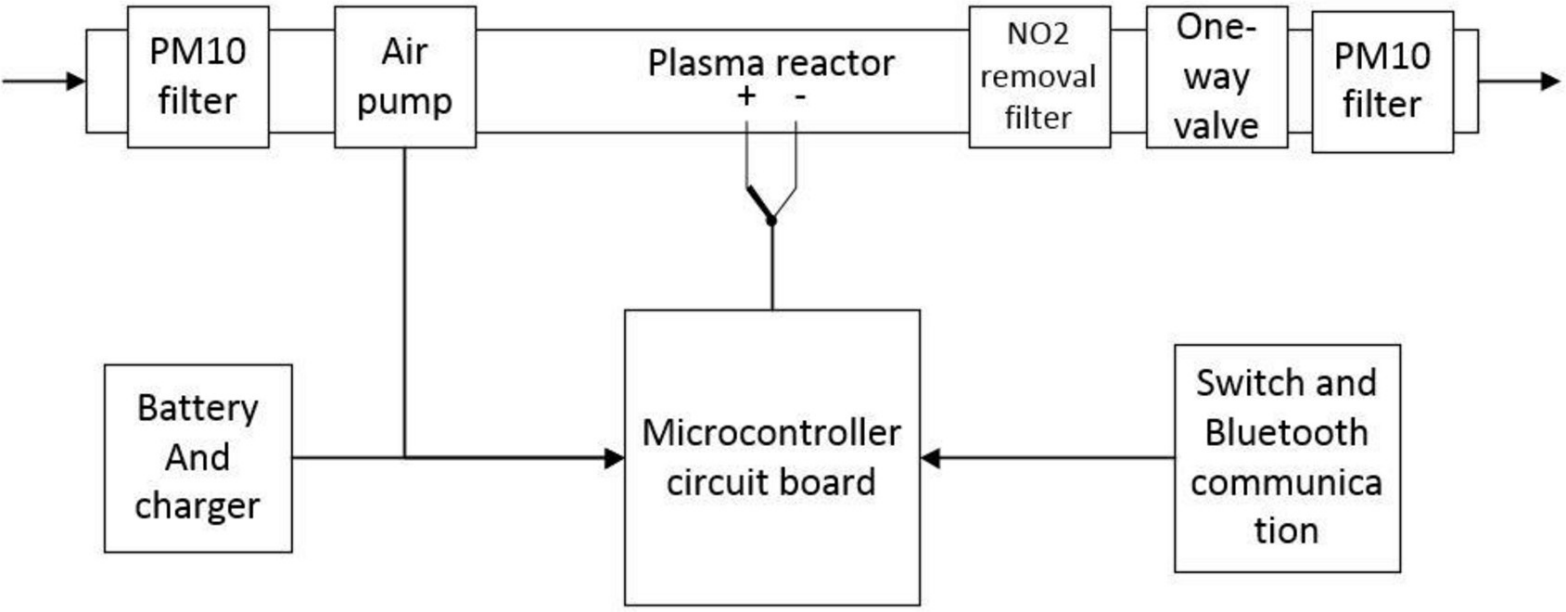
Schematic diagram of portable NO rescue device.

The N radical and O radical generated by ionization of N_2_ and O_2_ in dry air are combined to generate NO by pulsed discharge at atmospheric pressure. To begin with, a system that could maintain a continuous plasma reaction required development. A modulation module that could provide a breakthrough voltage and a continuous 5 kHz pulse maintenance voltage is shown in Fig 1. This system uses a 6000 mAh lithium battery as the energy source. The power manager uses a TP4056 chip to manage the charge and discharge of the battery. A microcontroller circuit board consists of an automatic buck-boost module and other control switches. The automatic buck-boost module is a combination of an XL 6019 boost module and LM2596S-ADJ buck module. The automatic buck-boost module provides an adjustable and stable power for the pulse generator and the air pump. The control switch is used to control the working status of each sub-module and to protect the safety of this system, and the Bluetooth communication module maintains the connection with the terminal equipment such as a cellphone.

**Fig 2:**
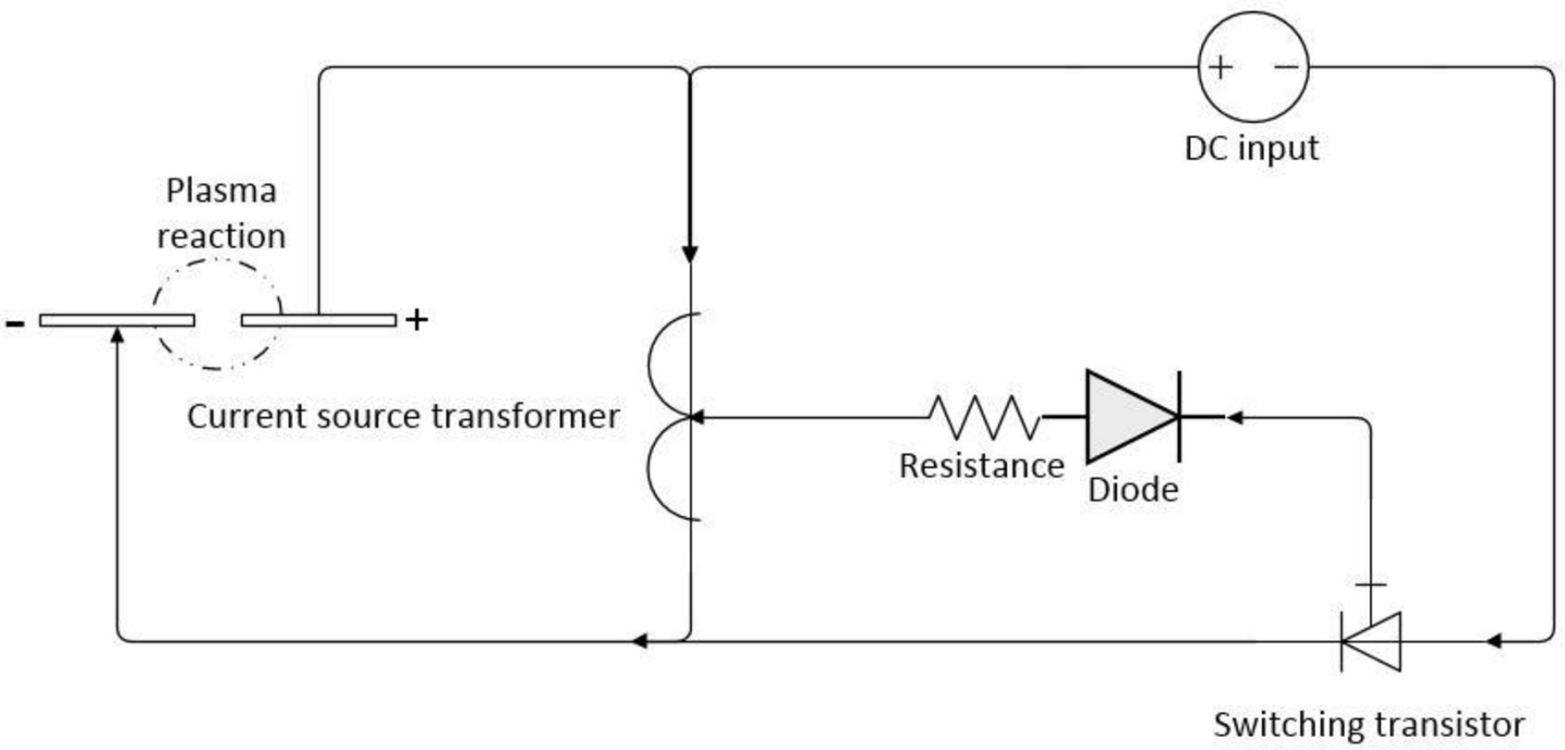
Schematic of pulse generator.

The pulse generator (Fig 2) is composed of a coupling coil, a switching transistor, a resistance, and a diode. The input voltage range is 3.8–32 V and the output voltage range is 1.25-35V. The pulse frequency from the switching transistor is about 12.5 kHz. This circuit could stably generate an arc discharge reaction with an input voltage of 3–7 V. After the boost of the coupling coil, the circuit could output a voltage up to but not higher than 15 kV, which provides the reaction voltage necessary for the plasma reaction chamber. The distance between the electrodes in the plasma reactor should not exceed 3 mm. Preliminary experiments showed that when the distance between the electrodes increased, the arc reaction required more power to stay stable. More violent reactions generated more NO, but the proportion of NO_2_ increased sharply at the same time. To ensure system stability, 1 mm distance between the electrodes was set to keep the reaction stable.

### Optimization experiment

**Fig 3:**
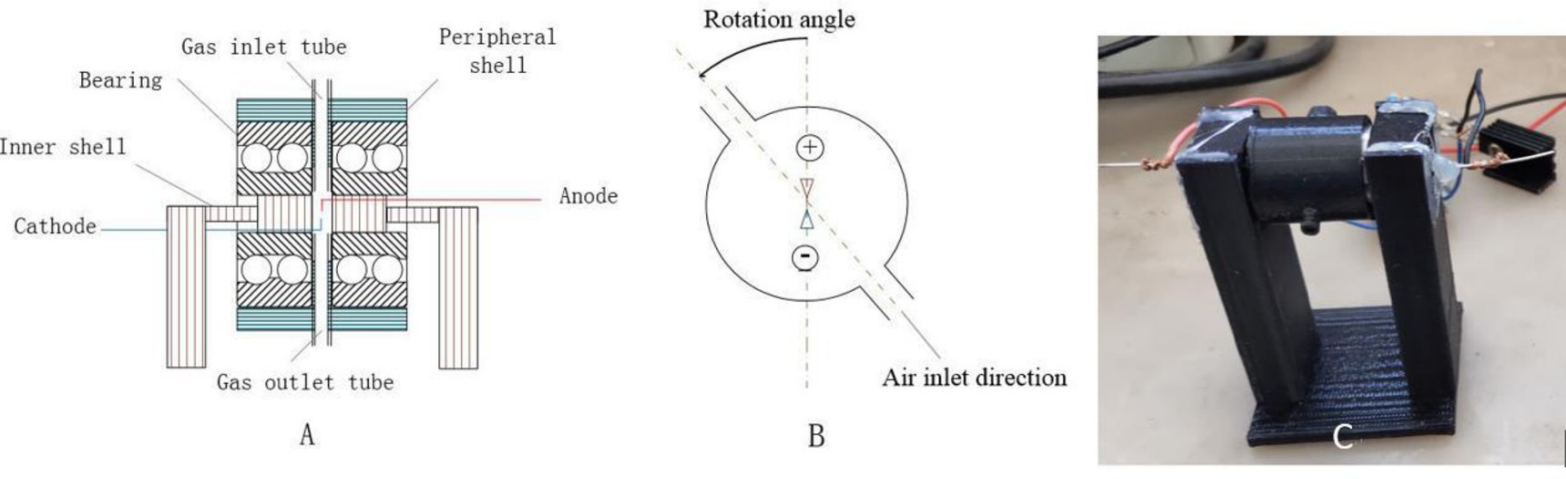
Structure of adjustable experimental reactor, A schematic diagram, B sketch map of direction angle between gas flow and discharging, C 3d print of adjustable experimental reactor.

To develop a more efficient plasma reaction chamber, an adjustable experimental reactor was made for an optimization experiment, the structure of which was composed of a reaction chamber and two electrodes as shown in Fig 3. The reaction chamber was a hollow cylinder with an inner diameter of 10 mm and a length of 20 mm. There were two symmetrical air holes in the center of the outer side of the cylinder, which are used for air inflow and outflow, respectively. A sealed bearing was installed on each side of the cylinder, and the center of the bearing was filled with sealing rubber. Two steel electrodes each with a diameter of 0.5 mm were passed through the sealing rubber, and bent into the shape shown in Fig 3A at the center of the hollow cylinder. The distance between the two electrodes was 1 mm. The stomata and the outer wall of the reaction chamber can be rotated 360° to explore the effect of airflow direction on the concentrations of NO and NO_2_. As shown in Fig 3B, the gas flowing from the cathode into the anode was defined as 0°, and counterclockwise was the positive direction. We made with 3d print technology as shown in Fig 3C.

A 520 motor-driven miniature air pump was employed in the optimization experiment, which provided 0.25–4 L/min gas supplies for the arc reaction system. Input voltage at 3–7 V was supported by the power source. A NO_2_ removal filter used Ca(OH)_2_ as the reducing agent to remove NO_2_ from NO. The removal filter was filled with loose Ca(OH)_2_ particles with a weight of 80g, which reduced NO_2_ to NO without generating other toxic or harmful gases. The PM10 filters fitted at both ends of the removal device dispersed the air flow and blocked particles in the air flow. An one-way valve was also fitted to protect the reactor from outside gas and unexpected mixture gas from a patient’s respiratory system.

The removal filter has a spiral tunnel tube to force the gas to flow a longer distance. The mixed gas of NO and NO_2_ can fully react with Ca(OH)_2_ particles with an equivalent length of about 250 mm, which can improve the removal of NO_2_ under a large gas flow effect.

The adjustable experimental reactor works as follows: the power switch turns on, and the plasma generator and plasma reactor then start to work. The electrodes in the reaction chamber create a stable arc, which generates NO from the air that the air pump supplies. The reaction gas flows through the NO_2_ removal filter to remove NO_2_, then the medical NO is exhausted. To develop a portable NO rescue device, three experiments were required to optimize the experimental parameters that affect the concentration of NO and NO_2_, including the adjustable design of airflow direction, gas flow rate, and input voltage.

### Measuring method

The gas flow rate was measured using a MF5706 flowmeter, which has a measurement range of 0 to 10 L/min and a resolution of 0.01 L/min. NO, NO_2_ and O_2_ concentrations were detected by a MultiRAE composite gas detector. The detector used electrochemical sensors to quickly detect the concentrations of NO, NO_2_ and O_2_. The maximum range was 250 ppm for NO, 20 ppm for NO_2_ and 30% Vol for O_2_. The resolution was 0.5 ppm for NO, 0.1 ppm for NO_2_ and 0.1% Vol for O_2_. The NO and NO_2_ concentrations were the average of the operating time of the reactor. In the experiment, some results exceeded the instrument range. To better measure the effect, some experiments used gas distribution to reduce the concentration before measuring.

## Results

The airflow direction, gas flow rate, and input voltage might have important effects on the generation of NO, NO_2_ and their ratio. The experiments were carried out around these three parameters to optimize the parameters of the portable NO rescue device. In the experiments, the electrodes were steel and the distance between the electrodes was 1 mm.

### Effect of airflow direction on arc plasma reaction

**Fig 4:**
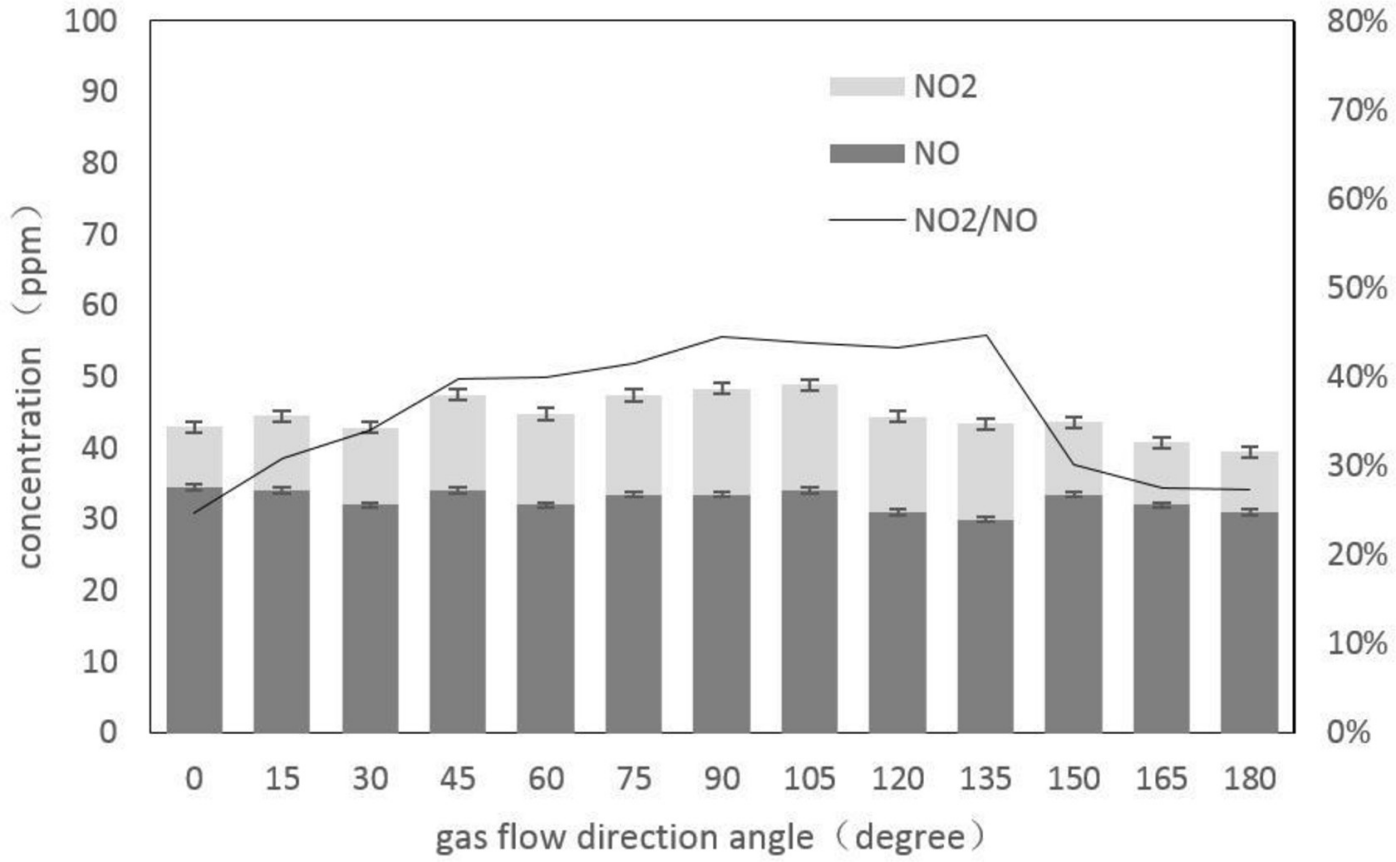
Effect of airflow direction on arc plasma reaction.

The angle between the gas flow direction and the arc was changed by the whirling of the reaction chamber. In the experiment, the gas flow angle from the cathode into the anode was 0°, and counterclockwise was the positive direction. Fig 4 shows the relationship between the concentrations of NO, NO_2_ and the gas flow direction changes when the gas flow is 2 L/min and the input voltage is 4 V.

As shown in Fig 4, the concentration of NO did not change much with the angle, only a slight increase or decrease. The concentration of NO_2_ first increased with the increase of the angle, and then gradually decreased. The concentration reached a higher level when the electrode was perpendicular to the airflow direction. The NO_2_/NO ratio increased first and then decreased as the angle increased. Airflow direction might effectively influence the NO_2_/NO ratio.

Next, the gas flow rate and input voltage were changed, and the gas flow rate and input voltage were set to 0.5 L/min, 3 V and 1.5 L/min, 3.5 V, respectively, and experiment repeated as above. The results obtained following these adjustments were consistent with those obtained above. When the angle was 0° which a higher concentration of NO might be obtained, while the concentration of NO_2_ was lower. As the 0° angle produced satisfactory flow direction results this was used as the selected angle in the subsequent experiments to optimize other parameters.

### Effect of gas flow and input voltage on arc reaction

**Fig 5:**
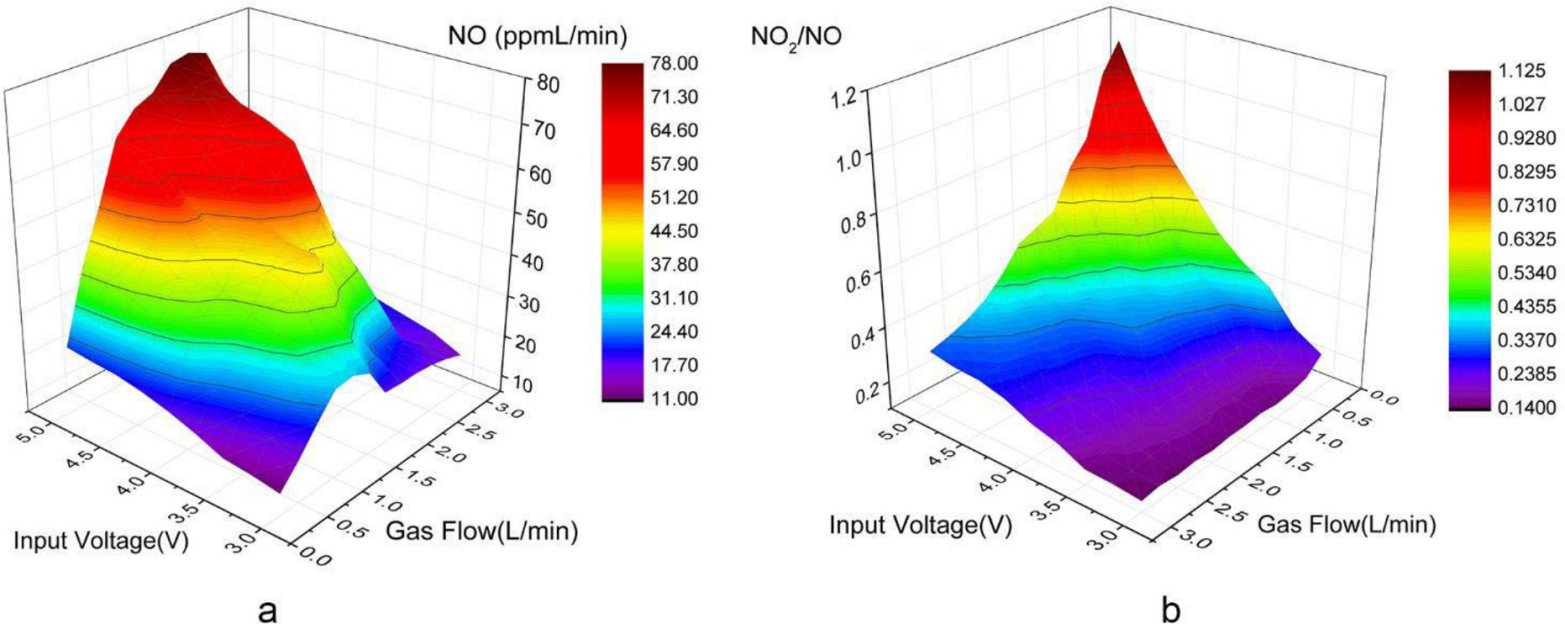
Effect of gas flow and input voltage on efficiency of NO production (a) and NO2/NO ratio (b).

In this experiment, gas flow capacity and input voltage were both taken into consideration. Results of the experiment are plotted in Fig 5. Fig 5A shows that with the increase of the gas flow rate, the amount of NO produced first increased and then decreased. This is because in an initial range the arc reaction was promoted by the increase in air flow. However, when the gas flow was too high, the arc reaction become unstable and NO could not be generated stably. When the airflow was 2 L/min, the total amount of NO reached or approached the maximum value under different input voltages (3.5–4.5V). When the airflow was constant, the higher the input voltage became, the greater the total amount of NO produced. As the voltage increased, the system power consumption and the amount of NO_2_ obviously increased, which was not conducive to the stable operation of the portable system. Therefore, the ratio of NO_2_ and NO was discussed.

The ratio of NO_2_ and NO was calculated during the experiment. For example, when the input voltage was 4 V, the reduction of NO_2_ concentration was more obvious following the increase of gas flow, and the NO_2_/NO ratio continued to decrease. When the gas flow rate was set as 2 L/min, the concentrations of NO and NO_2_ were increased as did the NO_2_/NO ratio. In the initial stage of increasing input voltage, the concentration of NO greatly increased, which then slowed with the subsequent voltage increases. Fig 5B shows the NO_2_ concentration increased slowly in the initial stage of voltage increase, and when the NO concentration increased slowly, the speed of the NO_2_ concentration increase increased. To obtain stable and high-quality NO, matching the gas flow rate and the input voltage was urgently required. High airflow and low voltage leads to low NO concentrations. In contrast, low airflow and high voltage leads to a risk of high NO_2_ concentrations. Combined with the actual needs of portable NO rescue devices, this system should maintain a good NO_2_/NO ratio while generating as much NO as possible. When the air flow was 2 L/min and the input voltage was 4V, an excellent output effect could be obtained.

### Filter effect of the NO_2_ removal device

**Fig 6:**
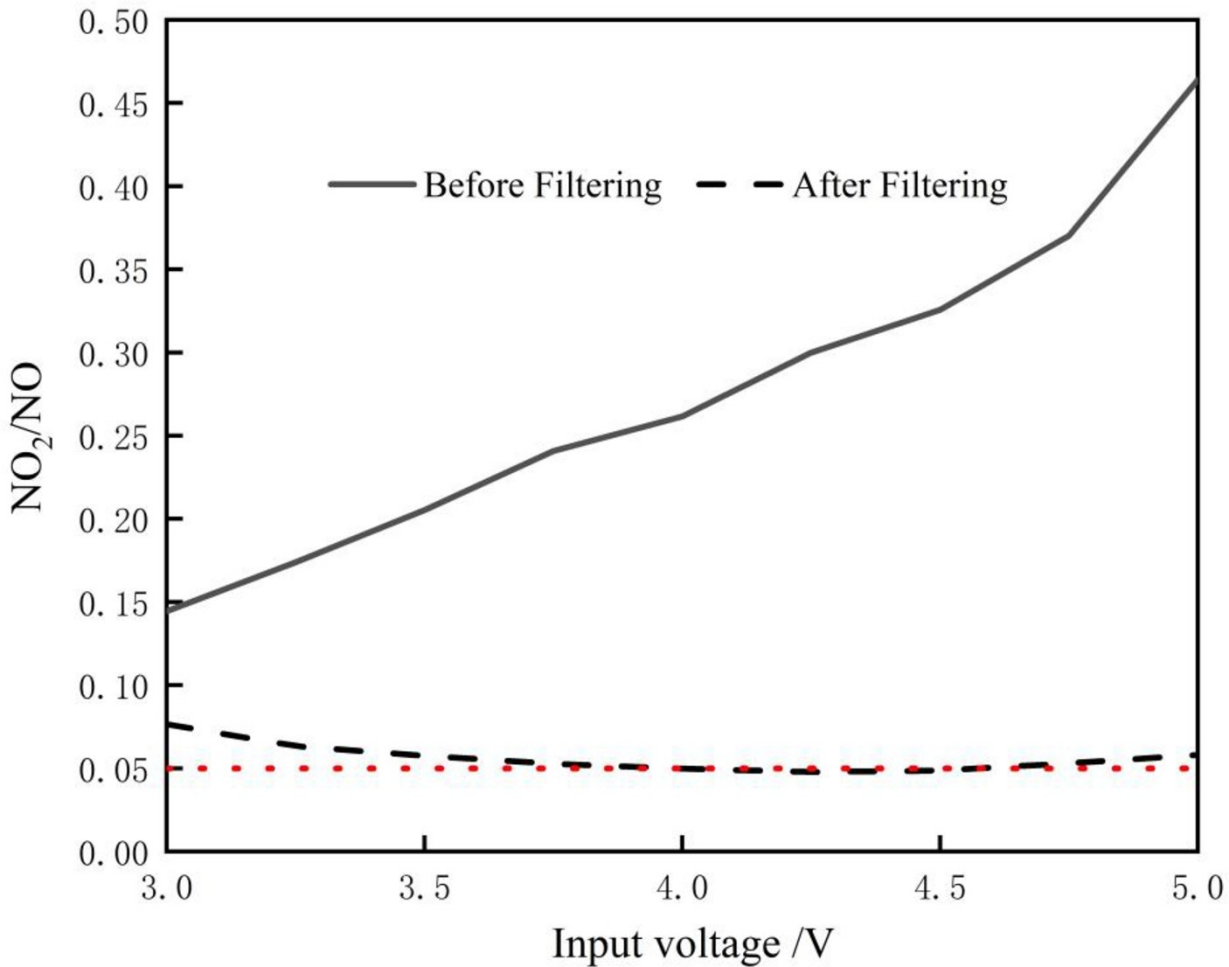
Variation of NO_2_/NO ratio with voltage before and after filtering.

After parameter optimization, the NO generator could stably produce at an air flow capacity of 2 L/min, concentration of NO at about 32.5±3.8 ppm. NO_2_ concentration was 8.5 ppm, and the NO_2_/NO ratio was 26%. However, this does not meet the medical requirement that the NO_2_/NO ratio should not exceed 5%. Therefore, the output gas needs to be filtered by a NO_2_ removal filter. The change in the NO_2_/NO ratio before and after filtering is shown in Fig 6. It can be seen that the NO_2_/NO ratio was greatly reduced by the obvious removal effect of Ca(OH)_2_ on NO_2._ This filter achieves a satisfactory filtering effect in the common working area of the NO generator (airflow 2 L/min, input voltage 3.5–4.5 V). NO_2_/NO ratio was maintained at about 5%, which meets medical requirements.

## Discussion

The aim of the study was to design and construct a portable NO rescue device, which could generate medical NO through high-frequency pulse discharge. High-frequency pulse discharge generates NO gas efficiently with a lower power consumption, which effectively solves the difficulties inherent in achieving a small and portable NO rescue device weighing less than 500g. The experimental system adopted a modular design with key parameters that could be easily adjusted and used to study the NO generation trends in the arc reaction. The experimental reactor was optimized, which improved the portable NO system in terms of airflow direction, gas flow rate, and input voltage on the concentration of NO, NO_2_ and NO_2_/NO ratio.

In the experiment, as the flow rate increased, the length of time in which the gas remained in the arc reaction zone decreased, the probability of N_2_ and O_2_ being ionized decreased, and the concentration of NO and NO_2_ also decreased. At the same time, as the reaction time decreased, the total production of NO_2_ decreasing was more obvious. It was also possible that as the gas flow rate increased, the temperature inside the plasma reactor decreased and the arc reaction rate slowed down, which resulted in the decrease of NO and NO_2_ concentrations. When the air flow increased to a critical value, the stability of the arc in the reaction chamber was significantly affected, and the arc reaction efficiency was obviously reduced.

Higher input voltage, more energy for arc reaction, lead to the increase in the degree of ionization of N_2_ and O_2_. The rate of NO and NO_2_ reactions increased with the increase of N radicals and O radicals. When the voltage was increased to a critical value, N radicals and O radicals were accumulated between the electrodes, thus the probability of generating NO_2_ was significantly increased. In other airflow states, the NO, NO_2_ concentrations and NO_2_/NO ratio had similar trends. However, as the airflow increased, the initial stage voltage also increased. Therefore, a further study of the purity and stability of generated NO was needed. The current work focuses on experimental research, and it is also necessary to study the impact of the discharge process on the generation of NO and NO_2_ in theory. The plasma density and temperature distribution can be analyzed by establishing an arc plasma simulation model. Establish a dynamic model of plasma chemical reaction to solve the arc reaction process. With some high-performance equipment to detect the plasma temperature. Adjust the NO and NO_2_ concentrations by controlling the plasma temperature.

The NO_2_ removal device used loose Ca(OH)_2_ particles as the reducing agent to remove NO_2_ from NO. The portable NO rescue device has a stable output of NO up to 32.5 ppm under 2 L/min airflow, which can be used in medical treatment with inhaled NO.

The portable NO rescue device still has some defects and deficiencies. For example, this paper mainly explored the regulation of NO and NO_2_ generation through experimental phenomena, including adjusting the experimental parameters and optimizing the structure of the reaction chamber. In the future, the discharge principle should be further studied, more key parameters such as discharge frequency and electrodes shape should be explored, and the NO generation ratio should be optimized. In addition, although Ca(OH)_2_ has a good filtering effect on NO_2_, the effect will be gradually exhausted. Some color reagents could be added into the NO_2_ removal device, which through their change in color over time could act as a prompt to the user to replace the aging filter device.

As a medical inhalable therapeutic gas, NO has not been widely used because it is difficult to produce and store. In this paper, a portable NO rescue device has been designed, which uses dry air at atmospheric pressure as a raw material to generate medical NO through high-frequency pulse discharge. As the device is small, portable, low cost and ready-to-use, the portable NO rescue device might have potential for use in epidemics involving diseases such as viral pneumonia by providing aided cannula therapy for critically-ill patients.

## Supporting information

S1 Table Example results of the airflow direction experiments for the different flow rates and voltages. (DOCX)

S1 Fig Concentration of NO and ratio of NO_2_/NO under 0.5 L/min gas flow and 3 V input voltage treatment. (DOCX)

S2 Fig Concentration of NO and ratio of NO_2_/NO under 1.5 L/min gas flow and 3.5 V input voltage treatment. (DOCX)
