# Supplementary material for: Design and study of nitric oxide portable producing device using continuous discharging arc plasma reaction keeping low energy efficiency for viral pneumonia emergency therapy
Source: PLoS One. 2020 Aug 13;15(8):e0237604. doi: 10.1371/journal.pone.0237604 (PMC7425952; doi:10.1371/journal.pone.0237604)
Supplement: S1 Table — (DOCX) [file pone.0237604.s001.docx]

**S1 Table. Example results of the airflow direction experiments for the different flow rates and voltages.**

|  | Gas Flow: 0.5L/min;  Input Voltage:3V | | | Gas Flow: 1.5L/min;  Input Voltage:3.5V | | |
| --- | --- | --- | --- | --- | --- | --- |
| Gas flow angle | NO | NO2 | NO2/NO | NO | NO2 | NO2/NO |
| 0 | 32 | 5.6 | 17.50% | 30 | 6.5 | 21.67% |
| 15 | 31 | 5.8 | 18.71% | 31 | 8.5 | 27.42% |
| 30 | 29.5 | 6.0 | 20.34% | 25 | 9.8 | 39.20% |
| 45 | 27 | 5.4 | 20.00% | 20.5 | 12.9 | 62.93% |
| 60 | 31 | 10.1 | 32.58% | 18.5 | 11.3 | 61.08% |
| 75 | 32 | 12.2 | 38.13% | 20.5 | 13.4 | 65.37% |
| 90 | 33 | 12.1 | 36.67% | 23 | 14.7 | 63.91% |
| 105 | 32 | 12.2 | 38.13% | 22 | 14.8 | 67.27% |
| 120 | 31 | 11.8 | 38.06% | 21 | 14.0 | 66.67% |
| 135 | 31 | 9.8 | 31.61% | 22.5 | 13.2 | 58.67% |
| 150 | 31 | 8.2 | 26.45% | 26 | 10.6 | 40.77% |
| 165 | 32 | 6.3 | 19.69% | 29.5 | 7.5 | 25.42% |
| 180 | 26 | 4.9 | 18.85% | 31 | 7.0 | 22.58% |
