# Supplementary figures and images for: Design and study of nitric oxide portable producing device using continuous discharging arc plasma reaction keeping low energy efficiency for viral pneumonia emergency therapy
Source: PLoS One. 2020 Aug 13;15(8):e0237604. doi: 10.1371/journal.pone.0237604 (PMC7425952; doi:10.1371/journal.pone.0237604)

**S1 Fig. Concentration of NO and ratio of NO2/NO under 0.5 L/min gas flow and 3 V input voltage treatment.**


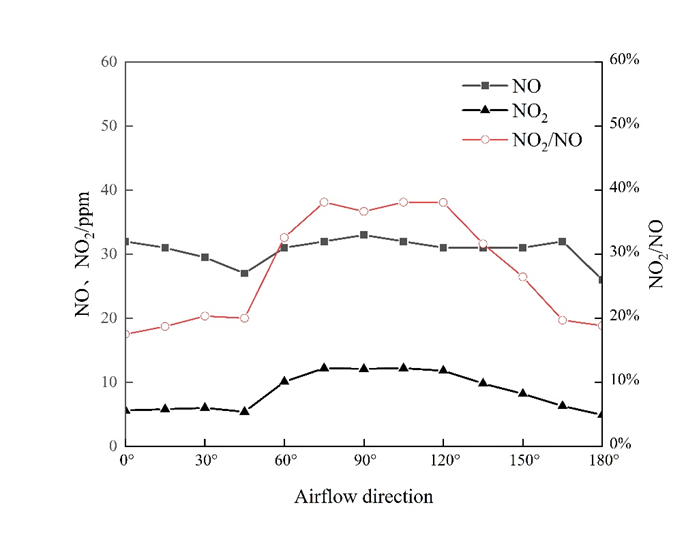

Supplement: S1 Fig — (DOCX) [file pone.0237604.s002.docx]

**S2 Fig. Concentration of NO and ratio of NO2/NO under 1.5 L/min gas flow and 3.5 V input voltage treatment.**


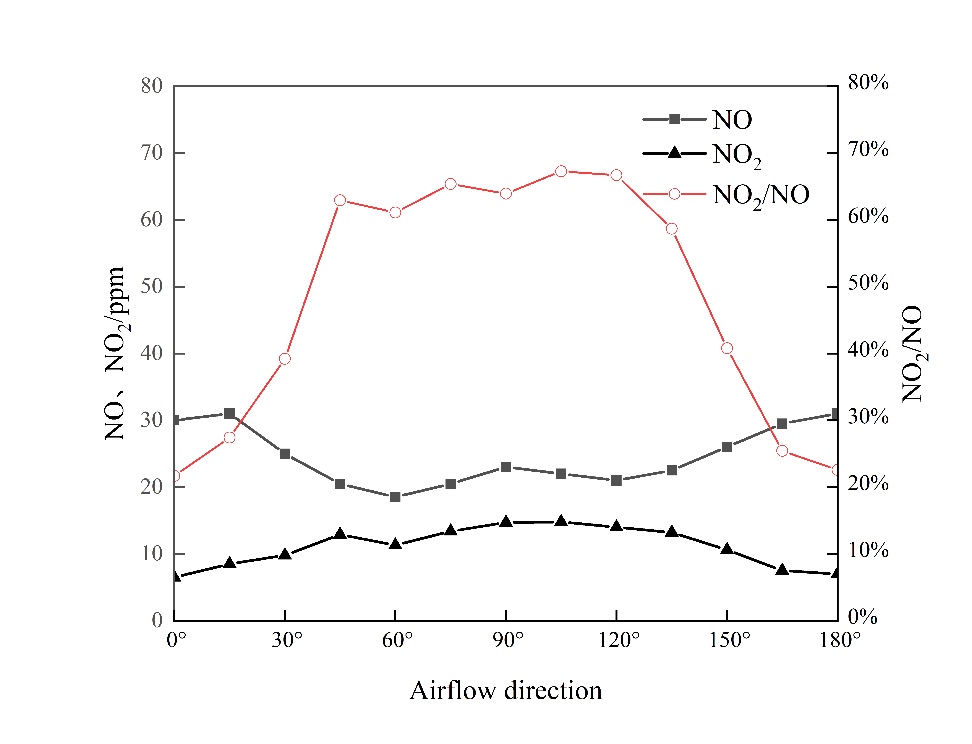

Supplement: S2 Fig — (DOCX) [file pone.0237604.s003.docx]
